# From Theory-Inspired to Theory-Based Interventions: A Protocol for Developing and Testing a Methodology for Linking Behaviour Change Techniques to Theoretical Mechanisms of Action

**DOI:** 10.1007/s12160-016-9816-6

**Published:** 2017-12-13

**Authors:** Susan Michie, Rachel N Carey, Marie Johnston, Alexander J Rothman, Marijn de Bruin, Michael P Kelly, Lauren E Connell

**Affiliations:** 1 Department of Clinical, Educational and Health Psychology, University College London, London, UK; 2 University of Aberdeen, Aberdeen, UK; 3 University of Minnesota, Minneapolis, MN, USA; 4 University of Cambridge, Cambridge, UK

**Keywords:** Behaviour change, Behaviour change techniques, Theory, Mechanism of action, Expert consensus

## Abstract

**Background:**

Understanding links between behaviour change techniques (BCTs) and mechanisms of action (the processes through which they affect behaviour) helps inform the systematic development of behaviour change interventions.

**Purpose:**

This research aims to develop and test a methodology for linking BCTs to their mechanisms of action.

**Methods:**

Study 1 (published explicit links): Hypothesised links between 93 BCTs (from the 93-item BCT taxonomy, BCTTv1) and mechanisms of action will be identified from published interventions and their frequency, explicitness and precision documented. Study 2 (expert-agreed explicit links): Behaviour change experts will identify links between 61 BCTs and 26 mechanisms of action in a formal consensus study. Study 3 (integrated matrix of explicit links): Agreement between studies 1 and 2 will be evaluated and a new group of experts will discuss discrepancies. An integrated matrix of BCT-mechanism of action links, annotated to indicate strength of evidence, will be generated. Study 4 (published implicit links): To determine whether groups of co-occurring BCTs can be linked to theories, we will identify groups of BCTs that are used together from the study 1 literature. A consensus exercise will be used to rate strength of links between groups of BCT and theories.

**Conclusions:**

A formal methodology for linking BCTs to their hypothesised mechanisms of action can contribute to the development and evaluation of behaviour change interventions. This research is a step towards developing a behaviour change ‘ontology’, specifying relations between BCTs, mechanisms of action, modes of delivery, populations, settings and types of behaviour.

## Teaser

The development of a formal method to link behaviour change techniques with mechanisms of action to support investigators as they design and evaluate behavioural interventions.

Human behaviour underlies many of the policy challenges of the twenty-first century: increasing health care needs, controlling epidemics and preventing pandemics, climate change, environmental degradation, poverty and inequality. In public health, behaviours (e.g. smoking, poor diet) contribute to many of the world’s leading causes of mortality including cardiovascular disease, lung cancer, stroke and HIV [1, 2] and contribute to approximately half of the premature deaths in Western societies [3]. Other behaviours protect health, such as appropriate care-seeking, attending medical screenings, accepting vaccinations and adhering to treatments. Further, the behaviours of health professionals in following guidelines, such as antibiotic-prescribing [4] and infection control [5], are hugely important [6].

Behavioural interventions have the potential to transform the health of populations, often at an extremely low cost [7, 8]. A number of effective interventions have a strong evidence base [9, 10]. Despite promising findings across a range of interventions, the systematic accumulation of evidence and guidance regarding how to develop effective interventions is slow. Findings from behaviour change interventions tend to be highly heterogeneous; the majority of interventions fail or have very minimal effects (see Cochrane database, e.g. [11–13]) and not at the scale required to bring about population-level changes.

The science of behaviour change has seen significant advances over the past few decades, including developing methods to standardise and improve the reporting of interventions and their underlying theory [9, 14–16]. However, for interventions to be effective, their active components (i.e. behaviour change techniques (BCTs)) should target relevant mechanisms of action. In this context, mechanisms of action are conceptualised as a range of theoretical constructs, defined broadly as ‘the processes through which a behaviour change technique affects behaviour’. It should be noted that not all theoretical constructs can be considered potential mechanisms of action (e.g. mechanisms of action in this context do not include personality traits, demographic variables or stages of change). Despite an abundance of BCTs and theoretical models of behaviour and behaviour change, we do not yet have an agreed-upon method for systematically linking BCTs to individual hypothesised mechanisms of action. This hinders the accumulation of evidence for how researchers hypothesise BCTs to have their effect.

## Behaviour Change Techniques: the Components of Behaviour Change Interventions

There is a recognised need to specify interventions in greater detail and with more consistent terminology and for evaluation studies to specify the components of the interventions in both the experimental and control groups [17–21]. In order to create a standardised vocabulary with which researchers and others can define and describe intervention components (i.e. *what* is delivered), Michie and colleagues, in collaboration with a large international network, developed a formal means for characterising behaviour change interventions, the BCT Taxonomy v1 (BCTTv1). This is an extensive, integrated, hierarchical classification system for reliably specifying intervention components in terms of 93 BCTs, organised into 16 groupings [15, 16, 22–24]. BCTTv1 incorporated a number of cross-behaviour BCT taxonomies [25, 26] and some behaviour-specific taxonomies for physical activity [27], alcohol use [28], smoking [29] and condom use [30].

BCTTv1 has been used to code interventions across a variety of behavioural domains, including physical activity and dietary behaviours [31, 32], oral hygiene behaviours [33], hazardous and harmful drinking [34], sexual health behaviours [35–37], blood pressure control/management behaviours [38], antibiotic-prescribing [39, 40] and diabetes preventative behaviours [41]. BCTTv1 has also been used by systematic reviewers to identify BCTs within intervention papers, in order to facilitate intervention comparison and evaluate technique efficacy (e.g. [42–47]). This is in line with recent guidance from the National Institute for Health and Care Excellence (NICE) which recommends that research should investigate which BCTs are effective in promoting the initiation and maintenance of behaviour change [9].

In addition to identifying individual BCTs that are effective in changing behaviour, research has noted the potential for groups of BCTs (i.e. theoretically related techniques) to work synergistically together. Assessments of published interventions have found that those that employed BCT groups were more effective than those that used only one BCT [48–50]. For example, interventions that combine self-monitoring with other self-regulatory BCTs (such as goal setting or action planning) have been associated with improved effectiveness [48].

Specification of intervention content by BCTs has transformed methods for reporting the content of behaviour change interventions, which facilitates greater precision and consistency in research [51]. However, a fuller understanding of intervention impact on behaviour and health requires knowledge of the mechanisms of action through which the BCTs have their effect. This has been recognised, for example, by the Cochrane Collaboration’s Effective Practice and Organisation of Care (EPOC) Group (e.g. [52]) and by NICE [53, 54]. Linking BCTs to the mechanisms of action described in behavioural theory allows researchers to target mechanisms of action more deliberatively and makes it easier for investigators to design studies that can evaluate the processes underlying effective interventions.

## Behaviour Change Theory: Specifying the Mechanisms of Action of Behaviour Change

At the heart of the science of behaviour change lies the pursuit of knowledge about the mechanisms of action through which behaviour change occurs. There is increased recognition of the need for systematic and extensive application of theory to the design of interventions [18, 55–59]; this is reflected, for example, in the UK Medical Research Council’s framework for designing and evaluating complex interventions [21] and the Intervention Mapping framework [60] for planning health promotion programmes.

Theories of behaviour change, which summarise what is known about constructs in the process of change, attempt to explain and predict when, why and how behaviour (change) occurs or does not occur, in addition to proposing both mechanisms of action and moderators of change along various causal pathways. A definition of theory from a multidisciplinary consensus exercise is: ‘a set of concepts and/or statements with specification of how phenomena relate to each other’, providing ‘an organising description of a system that accounts for what is known, and explains and predicts phenomena’ ([61], p. 5).

There are numerous formal theories which are generalisable across behaviours and/or contexts. These vary in complexity and range of application and many overlap with each other. A review led by psychologists, sociologists, anthropologists and economists identified 83 theories of behaviour and behaviour change, containing more than 1700 theoretical constructs [61, 62]. Given this abundance of theories, researchers and intervention designers are faced with difficult decisions as to which theory or theories they should draw on [63].

The Theoretical Domains Framework (TDF) was developed to address this challenge, aiming to make theories more usable and accessible to an interdisciplinary audience [64, 65]. The TDF specifies 14 theoretical domains, each of which includes several theoretical constructs that are similar in definition, but derive from different theories, and which may be relevant to understanding and changing behaviour. The TDF has been used in intervention development and design [66–68], as well as in systematic reviews [69–71]. Thus, whilst there are integrative frameworks such as the TDF, and general intervention development frameworks such as intervention mapping and others (e.g. [21, 60, 72, 73]), there is a need for a consensus about how the individual mechanisms of action specified in these theories can be linked with particular intervention components [18, 62].

## Linking BCTs to Mechanisms of Action

Developing ‘theory-based’ rather than ‘theory-inspired’ interventions, and understanding the theoretical basis for effective interventions, requires an understanding of links between BCTs and mechanisms of action. Preliminary work to address this has been conducted in both primary research and in evidence syntheses [9, 21, 26, 60, 74, 75]. Research in the UK has mapped a set of 35 BCTs to theoretically derived behavioural determinants [26]. Systematic literature reviews and meta-analyses have also examined the association between BCTs and theory [45], explored which BCTs are most effective in changing particular mechanisms of action, such as self-efficacy [76–79], and measured the overall effect of mechanisms of action, e.g. a change in intention on behaviour [80]. Research has also attempted to map BCTs to theoretical domains, which are clusters of theoretical constructs [22, 66], and to identify theoretical mediators of change using process evaluations [81–83]. Further, there are intervention development frameworks (e.g. the Behaviour Change Wheel Guide [72], Intervention Mapping [60] and the Theoretical Domains Framework [64, 65]), which offer guidance as to which BCTs to select for targeting mechanisms of action.

This work has demonstrated the potential for BCT-mechanism of action links to be identified. However, without a transparent, agreed-upon method for identifying hypothesised links, and a better understanding of the mechanisms of action believed to underlie each BCT, evidence accumulation will continue to be slow and unsystematic. In particular, there is a need to understand (i) how to operationalise ‘theory-based’ when designing interventions that draw on theory or theoretical constructs and (ii) how to make theoretical sense of interventions that specify BCTs used but without reference to theory.

**Fig. 1:**
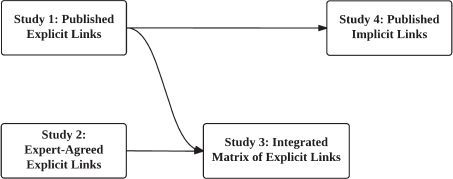
Flow diagram outlining the sequencing of studies in this project

The purpose of this research is to develop matrices of hypothesised links between BCTs and (i) specific mechanisms of action and (ii) behavioural theories. It will use two complementary data sources: published literature synthesis which encapsulates thinking in past peer-reviewed work and expert consensus which encapsulates current thinking (see Fig. 1for a flow diagram outlining the sequencing of studies in this project). We aim to identify explicit and implicit links that are made a priori within empirical studies of interventions and integrate them with links that are agreed on by experts in the field.Given that our interest is in examining the *thinking* of behaviour change researchers, as a step towards future research to empirically test these individual links, the links examined are hypothesised, rather than empirically tested. The matrices of links resulting from this initiative will contribute to the methodological resources available to behaviour change scientists, providing a more efficient and systematic way in which to identify and evaluate the theoretical processes hypothesised to underlie BCTs.

## Aims

1. To identify hypothesised links between intervention content (i.e. BCTs) and (i) mechanisms of action and (ii) behavioural theories
2. To make the results available as a resource to researchers and research users, especially behaviour change intervention developers

## Specific Objectives

1. Development: Identify how BCTs are hypothesised to link to mechanisms of action and how groups of BCTs are hypothesised to link to behaviour change theories. This will be achieved through (i) literature synthesis and (ii) consensus methods.
2. Evaluation: Produce a fully populated, integrated matrix of BCT-mechanism of action links and an additional matrix of BCT group-theory links, annotated to indicate the strength of the hypothesised links.
3. Implementation: Generate resources for applying the behaviour change matrices, including an online theory-based intervention manual that provides guidance on how to use the matrices in intervention design and evaluation.
4. Dissemination: Maximise the engagement of the international scientific community in the use and development of the behaviour change matrices through presentation and discussion of findings.

The sequence of the four studies in this project is outlined in Fig. 1.

## Study 1: Using Literature Synthesis to Examine Explicit Links Between BCTs and Mechanisms of Action in Published Interventions

### Aim

The aim of study 1 is to describe links between BCTs and their mechanisms of action, as explicitly hypothesised in published behaviour change interventions. We use the term ‘literature synthesis’ broadly to refer to the summarising of hypothesised BCT-mechanism of action links reported in a corpus of published intervention papers.

### Methods

In this study, we will first collate intervention development and evaluation papers in which links between BCTs and hypothesised mechanisms of action are explicitly stated and can be coded from the study reports. We will then evaluate these links by assessing the frequency, explicitness and precision with which they are reported. To maximise efficiency given the resource constraints of this 30-month project, this study will identify a corpus of literature in which hypothesised BCT-mechanism of action links are most likely to be present and codable.

### Procedure

#### Electronic Searches

Two approaches will be adopted to find studies reporting behaviour change interventions that include both BCTs and mechanisms of action. To optimally use resources to examine as many links as possible, the search strategies will prioritise papers that have already coded BCTs and/or identified mechanisms of action. As a first step, we will target papers in which BCTs have been coded using BCTTv1 or any of the taxonomies that were included in its development. This will be achieved through a forward search within the Web of Science and Google Scholar databases of five published BCT taxonomies [16, 25, 27–29]. Second, in order to allow us to efficiently screen interventions for mechanisms of action, we will identify reviews and interventions that have coded or outlined theory use (e.g. using the Theory Coding Scheme; [59]). To achieve this, we will forward search articles coded by a theoretical framework [59, 64, 65].

#### Contacting Experts

Researchers in the field of behaviour change will be contacted through mailing lists of several scientific and professional societies, including the Society for Behavioral Medicine, European Health Psychology Society, the UK Society for Behavioural Medicine and the Division of Health Psychology of the British Psychological Society. Members of the project’s International Advisory Board will also be contacted and asked to nominate interventions and reviews that have been coded by BCTs.

#### Review Reference Lists

The reference lists of reviews which have coded interventions by BCTs and/or theory will be checked and relevant papers downloaded. Where possible, the review authors’ original BCT and/or theory coding will be used to identify relevant interventions.

#### Inclusion Criteria

Peer-reviewed articles will be screened to ensure the paper is reporting a behaviour change intervention, and interventions will be included if authors have hypothesised that a particular BCT will have its effect on behaviour through a particular mechanism of action. Papers will be eligible for inclusion if the intervention content is detailed enough to identify BCTs and where the mechanism of action description is present and clear enough to allow a link to be identified. No restrictions will be made for year of publication, target behaviour, journal, quality of study or article type.

#### Data Extraction and Coding

Data will be entered into a relational database with two tables: (1) a ‘source’ table which contains all general information relating to the studies used to identify the links and (2) a ‘link’ table which contains details of all BCT-mechanism of action links identified. The tables will be connected using an identifying number. For the ‘source’ table, coders will extract all relevant general source information from intervention papers using a standardised data extraction sheet. Papers will be coded for author, year, intervention name (or other identifying feature of the intervention), article and study type, target behaviour and whether an underpinning theory/model of behaviour is mentioned. To inform other projects, additional general information will be extracted; full data extraction sheets are available in Appendices 1 and 2 (Electronic Supplementary Materials).

**Coding BCTs** BCTTv1 [16] will be used to code intervention descriptions. Where BCTs have previously been coded using an earlier version of the taxonomy, these will be re-coded using BCTTv1 to standardise coding.

**Coding BCT-Mechanism of Action Links** Each BCT-mechanism of action link will be coded according to the description within the intervention papers. To be coded as a BCT-mechanism of action link, the BCT must be hypothesised to change behaviour through one or more mechanism(s) of action. The label and definition of the mechanism(s) of action, as described by authors, will be extracted, and each link will be given a unique row within the link table. The number of specific BCT-mechanism of action links will represent the frequency of these links in the scientific literature. In addition to frequency, we will code the explicitness of each link as either 1 = not explicit (some inference needed) or 2 = very explicit (no inference needed). We will also code whether the link is described as individual (i.e. one BCT to one mechanism of action) or grouped (i.e. more than one BCT linked to one mechanism of action or one BCT linked to multiple mechanisms of action), as well as whether the link is empirically tested. These two additional dimensions were included based on initial pilot work to develop coding guidelines. This work indicated that there was likely to be wide variation in the explicitness and precision of the links. Although there are many other interesting dimensions that could be included (e.g. whether the link was based on theory, previous research and/or authors’ own perspectives), the constraints of the project preclude us from including these additional features. At the end of the project, the study 1 coding database will be made publically available, and it will be possible for us and others to conduct additional analyses of data from these studies.

**Reliability Analysis** Inter-rater reliability between coders will be calculated for both BCTs and BCT-mechanism of action links. Reliability will be calculated using Prevalence and Bias Adjusted Kappa PABAK [84], as has been used in previous BCT coding research to allow for the high prevalence of negative agreement and differences between coders in their threshold for coding a BCT [15].

#### Data Analysis

The analysis will examine hypothesised links between BCTs and mechanisms of action. The frequency of each BCT-mechanism of action link will be represented in graphical form on a heat map (for an example, see: https://en.wikipedia.org/wiki/Heat_map). We will also examine the explicitness (i.e. whether or not the link coding required inference) and precision (i.e. the extent to which a BCT links to one mechanism of action above other mechanisms of action) of each link. The following questions will be addressed through the analysis.

Based on hypothesised links within the published intervention literature:

1. How frequently is BCT X linked to mechanism of action Y?
2. Which BCTs are most frequently linked to mechanism of action Y?
3. Which mechanisms of action are most frequently linked to BCT X?

Additionally, we will explore whether any links occur more often than might be expected given the frequency of occurrence of each BCT and mechanism of action.

## Study 2: Examining Explicit BCT-Mechanism of Action Links Through Expert Consensus

### Aim

Alongside investigating the explicit links within published interventions in study 1, which encapsulates thinking in previous peer-reviewed work, study 2 will use expert consensus methods to elicit and encapsulate current thinking of behaviour change experts.

### Methods

#### Participants

Participants will be 105 expert judges with extensive experience in designing, evaluating and/or synthesising evidence about theory-based behaviour change interventions. The 105 experts will be separated into five groups (21 experts per group). These numbers were selected based on previous expert consensus studies and an initial pilot of the task.

Given the importance of including a diverse sample of experts from a broad range of disciplines and backgrounds, we will recruit experts from a variety of scientific and applied fields (e.g. academic psychologists, practising clinicians, social scientists working in the voluntary/community and commercial sectors). The experts will be recruited via email from those who participated in either online BCT Taxonomy training (http://www.bct-taxonomy.com/), training workshops or the BCT Taxonomy v1 project ([15]; http://www.ucl.ac.uk/health-psychology/bcttaxonomy), members of the project’s International Advisory Board and scientific and professional societies and centres (UCL’s Centre for Behaviour Change, the Special Interest Group of the US Society for Behavioral Medicine, European Health Psychology Society, UK Society for Behavioural Medicine and Division of Health Psychology of the British Psychological Society). We will also ask for recommendations for expert judges from those already recruited.

Individuals who express an interest in becoming an expert judge will be asked to complete a self-assessment questionnaire (see Electronic Supplementary Materials Appendix 3) to evaluate their relevant expertise. Previous research has indicated that participants’ self-rated expertise is a predictor of initial accuracy of judgement in consensus exercises [85]. Included in the study will be those who (i) rate their expertise in BCTs, behaviour change theories and interventions as ≥4 (on a 7-point scale, where 0 indicates ‘no expertise’ and 7 indicates ‘profound expertise’) *and* (ii) report that they have designed or helped to design a behaviour change intervention(s) that ‘used specific behaviour change techniques’ and that ‘was specifically grounded in a behaviour change theory/ theories’ at least ‘to some extent’. If more than 105 people with the relevant expertise are recruited, we will select a sample to reflect a range of academic disciplines, professions and countries, following recommended practice in constructing expert panels [86].

### Procedure

#### Consensus Development Method

A formal consensus development method drawing on Nominal Group Technique (NGT) [87] will be used. The NGT follows explicit steps that can be replicated, is commonly used and is a feasible and reliable technique for ranking sets of proposals [88]. NGT depends on two-way, iterative information exchange, using a basic Delphi structure and a discussion component in which participants can share and reflect on their perspectives. NGT typically takes place face-to-face and can be cost- and time-intensive [86]. Our modified NGT will take place online [89], with expert ratings given via Qualtrics [90] and the discussion hosted on an online forum called ‘Loomio’ [91]. This type of computer-mediated communication allows for structured interaction in distributed groups [86] whilst retaining the basic principles of NGT.

#### Identifying the Mechanisms of Action to Be Studied

The set of mechanisms of action considered in this study will be (i) the 14 theoretical domains as described in the Theoretical Domains Framework (TDF) [64] and (ii) the 12 most frequently occurring mechanisms of action derived from a set of 83 behaviour change theories [62]. Each of the theoretical domains of the TDF contains several related theoretical constructs identified previously via consensus methodology, whilst the 12 frequently occurring mechanisms of action are additional single constructs extracted from a review of 83 theories. These were included to ensure broad coverage of the potential mechanisms of action. Thus, the set of mechanisms of action for this study will be restricted to these 26. The restriction reflects the need to minimise burden and maximise feasibility of the task for expert participants as well as the resources available to conduct the study.

#### Conduct of the Consensus Exercise

For the task, experts will consider the 61 most frequent BCTs, i.e. those identified more than twice, in 40 systematically selected and coded descriptions of interventions across a range of contexts [15]. In piloting the consensus task, 13–14 BCTs × 26 mechanisms of action per expert was found to be feasible. Expert panels of 20 or more members have demonstrated stability in previous consensus studies [92]. Thus, in order to evaluate hypothesised links between all 61 BCTs and 26 mechanisms of action, 5 groups of approximately 20 experts are needed. Experts will be randomised to one of 5 groups, with 21 experts per group. BCTs will be rank-ordered according to their frequency of use in interventions [15] and these will be assigned to groups of experts using stratified random allocation. In addition, all groups will be asked to review the two most frequent BCTs to assess similarity across groups and the appropriateness of between-group analyses. In total, the 5 groups of experts will be asked to consider possible links between these 13 or 14 BCTs and the 26 mechanisms of action (see Table 1 of Electronic Supplementary Materials).

In round 1, participants will be sent their set of BCTs and mechanisms of action, with definitions of both. In an online questionnaire [90], each BCT will appear individually and participants will be asked to judge whether that BCT changes behaviour through a particular mechanism of action, on a 5-point scale (definitely no, probably no, do not know/uncertain, probably yes, definitely yes). Experts will be instructed to focus on the ‘key’ mechanisms of action they believe a BCT might change in order to change behaviour. We will acknowledge that other variables may be implicated in the processes but ask them to identify what they consider to be the key mechanisms of action. The order of the 26 mechanisms of action that appear on screen will be randomised. This rating task will be completed for each BCT. Following round 1, each group of 21 participants will be emailed a statistical summary (i.e. frequency distributions) of their group’s responses from round 1 ratings, as well as a reminder of their own responses.

In round 2, experts will participate in an online discussion, hosted via *Loomio*. Research shows that providing participants with feedback about the reasons for group responses (e.g. through discussion), in addition to statistical feedback, leads to the greatest improvement in accuracy over rounds [85]. The purpose of the discussion round will be to exchange views about the BCT-mechanism of action links, focusing on those for which there was high uncertainty (i.e., a high proportion of ‘do not know/uncertain’ responses) and disagreement (e.g. similar number of probably/definitely no and probably/definitely yes responses). The online discussion will take place within each of the five expert groups and will be anonymous. Anonymity will be ensured by asking participants to sign into the discussion using a pre-assigned expert identification number (e.g. ExpertB12) and not their name. The discussion will be asynchronous to facilitate experts participating from various time zones. Online, asynchronous discussions have been found to be an efficient means of communication in this context [86, 93]. There will be anonymous discussion moderators from the research team who will periodically summarise the discussion and raise issues for further consideration.

In round 3, participants will receive the same BCTs and mechanisms of action as in round 1 and be asked to provide new ratings (which may or may not be the same). They will have access to summary ratings, in graphical form, of their group’s responses from round 1 and will also have access to the transcripts of their group’s round 2 discussion. This information will be provided in order to allow experts to reevaluate their original ratings in light of the results and, if they find the composite group ratings more convincing [94], adjust their responses accordingly. For this final round, experts will be asked ‘When BCT X works, does it work through changing: [list of mechanisms of action]’. The response options for the final round will be ‘definitely yes’, ‘definitely no’, ‘uncertain’ and ‘possibly’.

#### Data Analysis

Round 3 ratings of the links between 61 BCTs and 26 mechanisms of action will be categorised to examine agreement. There will be four categories: agreement that there is a link, agreement that there is no link, disagreement, and uncertainty.

The following questions will be addressed through the analysis:

1. What proportion of experts believe BCT X is linked to mechanism of action Y?
2. Which BCTs are most linked to mechanism of action Y by experts?
3. Which mechanisms of action are most linked to BCT X by experts?

Additionally, we will explore whether a greater proportion of experts rated certain BCTs and mechanisms of action as ‘definitely’ linked more than might be expected given the proportion of ratings for each BCT and mechanism of action.

## Study 3: Triangulation of Consensus and Literature Synthesis Methods

### Aim

Studies 1 and 2 will provide evidence about hypothesised links between BCTs and mechanisms of action. Study 3 aims to (i) evaluate the agreement between the matrices from the literature synthesis of study 1 and expert consensus of study 2 and produce an integrated matrix of BCT-mechanismof action links, annotated to indicate strength of evidence produced by studies 1 and 2 (see Fig. 1).

### Methods

#### Participants

Approximately 20 experts who design, evaluate and/or synthesise evidence about theory-based behaviour change interventions, who have not participated as expert judges in study 2, will be recruited for this study. The new experts will be recruited via the project’s International Advisory Board, through email (as in study 2), as well as through mailing lists of several professional and scientific societies. A self-assessment questionnaire, similar to that used in study 2, will be sent to individuals interested in participating. If more than the required number of experts are interested in participating, we will select the experts with the highest scores relating to experience with, and knowledge of, BCTs and behaviour change theories.

#### Procedure/Analysis

We will first investigate the extent to which the evidence for links identified by study 1 is comparable to that identified by study 2. Then, the agreement between the two data points (one from each study) for each BCT-mechanism of action link will be evaluated by participants. Thus, experts will draw on the findings of literature synthesis, expert consensus and their own knowledge and expertise. Where there are discrepancies between the evidence from the literature and expert opinions, possible reasons for discrepancies will be elicited and attempts to reach consensus amongst the experts will be made using the methods used in study 2 (modified NGT and, if needed, a Webinar). A matrix of consensus-based and literature-based hypothesised BCT-mechanism of action links, annotated to indicate strength of evidence, will be generated as the output from this study.

The following question will be addressed through the analysis: How does the evidence from studies linking BCT X to mechanism of action Y compare to the evidence from experts rating this link?

## Study 4: Examining Links between Groups of BCTs and Implicit Theories

### Aim

The frequency with which group of co-occurring BCTs are used in interventions may reflect the extent to which there is a shared, often implicit, theorising of a synergistic or additive relationship between BCTs. The objective of study 4 is to identify whether these group of BCTs can be linked to specific theories. This study has two phases: grouping of BCTs within interventions from the study 1 literature synthesis, and expert consensus.

### Methods

#### Participants

Approximately 20 experts who design, evaluate and/or synthesise evidence about theory-based behaviour change interventions, and who collectively have knowledge of a wide range of theories, will be recruited. Experts from studies 2 and 3 will be invited to participate in this study. Given the high level of knowledge of behaviour change theories that will be needed for this study, priority will be given to those who were active in previous discussions and demonstrated advanced knowledge of behavioural theories.

### Procedure

#### Literature Synthesis

The peer-reviewed intervention reports from study 1 will be used to identify the extent to which there are groups of BCTs that group together in interventions. This will be a data-driven approach to identifying implicit BCT groups.

#### Consensus Exercise

Next, a four-round consensus method will be adopted. In an initial round (round 1), expert judges will complete an open response task, listing theories that might underlie each group of BCT. To facilitate this process, the experts will be given an overview document of theories that they can consult, and they can also suggest other theories not covered in this list.

In round 2, the same experts will be presented with the same BCT groups as well as the list of theories identified as relevant for each group in the initial round. Each BCT group will appear individually on screen and experts will be asked to judge whether the BCT groups links to a particular theory. The order of theories groups that appear on screen will be randomised.

Similar to study 2, this will be followed by an online discussion in which experts can anonymously exchange views about the links, focusing on those links for which there was high disagreement and uncertainty (round 3). A final round (round 4) will ask experts to re-evaluate their original ratings and provide new ratings (which may or may not be the same), with reasons detailing their decisions. In addition, experts will be asked to comment on a future research agenda in this area. These final ratings will be used in all analyses. The consensus exercise will be hosted online [90], as in study 2.

#### Data Analysis

The extent to which BCTs group within these published interventions will be identified. Experts’ round 4 data for each link between BCT groups and theories will be categorised to examine agreement. Categories will include agreement that there is a link, agreement that there is no link, disagreement, and uncertainty. Data will be analysed to address the following questions:

1. To what extent are there BCTs that group together in interventions?
2. Which BCT groups are most linked to theory Y by experts?
3. Which theories are most linked to BCT group X by experts?

### Output

The output of studies 1 and 2 will be matrices of BCT-mechanism of action links, whose cells will denote frequency of links and proportion of experts rating links, respectively. Study 3 will produce an integrated matrix representing the converging evidence of studies 1 and 2. Study 4 will produce a matrix of BCT group-theory links along with the extent of agreement for the link for each cell of the matrix. If there is no consensus for specific links, this will be reported.

### Discussion

The development of an initial matrix of BCTs and mechanisms of action with links to behavioural theories, and the methodology for creating this matrix, will contribute to the science of behaviour change in a number of ways. First, agreed links between BCTs and mechanisms of action and theories will support possibilities for testing and refining existing theories, and advancing our understanding of how interventions have their effects on behaviour. Examining the thinking of behaviour change researchers will be an important step towards building a matrix of hypothesised BCT-mechanism of action links for future empirical testing and experimental research. Findings from this research are not intended to be final; rather, the aim is that they will generate hypotheses for future empirical studies. Second, it will produce a resource to aid in the development of theory-based interventions and/or analysis of published evaluations in terms of their mechanisms of action. This would constitute an important contribution to the methodological resources available to complex intervention researchers. Third, it will help elucidate the deeply embedded and explicit and implicit theories which are present in behaviour change thinking and enable them to be linked to other theories used in the behavioural and social sciences. This will help in the analysis of the ontological and epistemological divergences and similarities in the social sciences [95]. Finally, this work will contribute to the bigger vision of developing an ‘ontology’ of behaviour change (see www.humanbehaviourchange.org), specifying relationships between BCTs, mechanisms of action, modes of delivery, populations, settings and types of behaviour, as a foundational step for developing more effective behaviour change interventions.

### Limitations

Despite the above contributions, a number of potential limitations of this work should be noted. First, as outlined, this research aims to identify the explicit and implicit links that are *hypothesised* in published interventions and by behaviour change experts. Thus, we will make no inferences regarding the empirically demonstrated statistical associations of these links. Second, given the resource and time constraints of the project, our literature synthesis research sets out to identify interventions in which authors hypothesise BCT-mechanism of action links. Study 1 will not be a systematic review and the included studies may not be representative of all published behaviour change interventions. Third, whilst the proposed analysis of study 1 data will allow us to examine BCT-mechanism of action links explicitly hypothesised by authors, one cannot assume that the links not included in these papers reflect a belief that these BCTs and mechanisms of action are not linked. Rather, this may be due to the wording in the paper or an omission of detail by the authors. Finally, all of the consensus procedures depend on the selection of experts and the results therefore depend on their willingness to participate and the extent to which their opinions are representative of the wider behaviour change research community.

### Dissemination and Implementation

We will increase awareness and understanding of the hypothesised BCT-mechanismof action and BCT-theory links through dissemination of the work and implementation of the findings. We will develop a guide to developing and evaluating theory-based interventions, providing wide access to the multidisciplinary research and user community [in similar fashion to those produced for the BCT Taxonomy project [15, 16, 23, 24]; see also http://www.bct-taxonomy.com]. The resource will include the study outputs, worked examples of applying the matrices to complex intervention design and evaluation and research recommendations.

There will be a facility for commenting on our methodology and findings, for reporting experiences of applying the links and for sharing relevant research. We will link this with both the current project website and with the US National Institute of Health’s Grid-Enabled Measures (GEM) portal (see https://www.gem-measures.org/Public/Home.aspx). We will hold national and international multidisciplinary workshops to introduce the developed matrices and provide supervised experience using the distance learning tutorial system, as developed for the BCT Taxonomy study. Overall, findings will be reported via national and international conferences, relevant high-quality peer-reviewed journal articles, presentations in association with MRC’s regional hubs of excellence and Population Health Sciences Research Network, and via the study website, which will also contain links to relevant sites.

### Building the Consensus

In order to develop maximum international consensus around this project, we have established an International Advisory Board comprising 42 researchers from 10 countries http://www.ucl.ac.uk/behaviour-change-techniques/People/iab. We will also liaise with relevant groups and societies, including the US Society Behavioral of Medicine’s Special Interest Groups and the European Health Psychology Society.

## Ethics, Research Governance and Data Preservation for Sharing

The study will conform to relevant ethical and legal guidelines for participant consent, confidentiality and data storage. All data will be preserved and its availability for use by other research teams will be publicised via the website resource, on the Open Science Framework (see https://osf.io/), and as part of our dissemination work.We will institute an automatic registration system to track usage of this database.

## Supplementary Material

Supplementary Materials
