# Supplementary material for: From Theory-Inspired to Theory-Based Interventions: A Protocol for Developing and Testing a Methodology for Linking Behaviour Change Techniques to Theoretical Mechanisms of Action
Source: Ann Behav Med. 2017 Dec 13;52(6):501–12. doi: 10.1007/s12160-016-9816-6 (PMC6367898; doi:10.1007/s12160-016-9816-6)
Supplement: Supplementary Materials [file s12160-016-9816-6_suppl_supplementary_materials.pdf]

Supplementary Materials

Table 1: List of 26 mechanisms of action included in Study 2

	Mechanism of Action Definition	Mechanism Label
1.	An awareness of the existence of something	Knowledge
2.	An ability or proficiency acquired through practice	Skills
3.	A coherent set of behaviours and displayed personal qualities of an individual in a social or work setting	Social/ Professional Role and Identity
4.	Beliefs about one's ability to successfully carry out a behaviour	Beliefs about Capabilities
5.	Confidence that things will happen for the best or that desired goals will be attained	Optimism
6.	Beliefs about the consequences of a behaviour (i.e. perceptions about what will be achieved and/or lost by undertaking a behaviour, as well as the probability that a behaviour will lead to a specific outcome)	Beliefs about Consequences
7.	Processes by which the frequency or probability of a response is increased through a dependent relationship or contingency with a stimulus or circumstance	Reinforcement
8.	A conscious decision to perform a behaviour or a resolve to act in a certain way	Intentions
9.	Mental representations of outcomes or end states that an individual wants to achieve	Goals
10.	Ability to retain information, focus on aspects of the environment and choose between two or more alternatives	Memory, Attention and Decision Processes
11.	Aspects of a person's situation or environment that discourage or encourage the behaviour	Environmental Context and Resources
12.	Those interpersonal processes that can cause oneself to change one's thoughts, feelings or behaviours.	Social Influences
13.	A complex reaction pattern involving experiential, behavioural, and physiological elements	Emotion
14.	Behavioural, cognitive and/or emotional skills for managing or changing behaviour	Behavioural Regulation
15.	The attitudes held and behaviours exhibited by other people within a social group	Norms
16.	One's *perceptions* of what most other people within a social group believe and do	Subjective Norms
17.	The general evaluations of the behaviour on a scale ranging from negative to positive	Attitude towards the behaviour

18.	Processes relating to the impetus that gives purpose or direction to behaviour and operates at a conscious or unconscious level	Motivation
19.	One's conception and evaluation of oneself, including psychological and physical characteristics, qualities and skills	Self-image
20.	Deficit of something required for survival, well-being or personal fulfilment	Needs
21.	Moral, social or aesthetic principles accepted by an individual or society as a guide to what is good, desirable or important	Values
22.	Processes through which current behaviour is compared against a particular standard	Feedback Processes
23.	A process by which thoughts, feelings and motivational states observed in others are internalised and replicated without the need for conscious awareness	Social Learning / Imitation
24.	Processes by which behaviour is triggered from either the external environment, the performance of another behaviour, or from ideas appearing in consciousness	Behavioural Cueing
25.	Evaluations of an object, person, group, issue or concept on a scale ranging from negative to positive	General Attitudes/ Beliefs
26.	Perceptions of the likelihood that one is vulnerable to a threat	Perceived susceptibility/ vulnerability

Appendix A

Study 1 Published Explicit Links: Source Data Extraction Sheet

**Source ID**	**Researcher initials**	**Author**	**Year**	**Intervention name**	**Article type**	**Study type**	**Target behaviour**	**Theory mentioned**	**Intervention theory / theories**	**Intervention development framework**	**BCT coded**
				Name or identifying feature of intervention (to avoid including multiple papers reporting same intervention)	1= Outcome evaluation<br>2= Process evaluation<br>3= Protocol<br>4= Intervention development / description	1= RCT<br>2= Cluster RCT<br>3= Randomised trial<br>4= Pre/post test<br>5 = Quasi-experiment 6 = Pilot<br>7 = Feasibility<br>8 = Field experiment	Add in words	0= No theory mentioned<br>1= Theory mentioned, unclear if underpins intervention<br>2= Theory guided intervention	List theory or theories only if clear it guided intervention	0 = None or Unclear<br>1 = Intervention Mapping<br>2 = Precede Proceed<br>3 = BCW<br>4 = MRC framework	BCTs identified by author(s) in paper using taxonomy

Appendix B

Study 1 Published Explicit Links: Link Data Extraction Sheet

**Source ID**	**Researcher initials**	**BCT**	**BCT confidence**	**MoA**	**MoA definition**	**Target behaviour**	**Explicitness**	**Grouped**	**Empirically tested**	**Page**	**Comments**
		Number as per Coding Guidelines	1 = present in all probability 2 = present beyond all reasonable doubt		Add definition from paper where possible	Add in words	1 = BCT hypothesised to change behaviour through mechanism but some inference needed 2 = BCT explicitly hypothesised to change behaviour through mechanism	1 = 1 BCT linked to 1 MoA 2 = Group of BCTs explicitly linked to one mechanism or one BCT linked to group of mechanism	0 = No 1 = Mechanism measured 2 = BCT-mechanism link tested	Add page number where link is specified	Copy and paste relevant text from paper

Appendix C

Study 2 Expert Agreed Explicit Links: Expert Recruitment Self-Evaluation Form

1. To what extent have you:

(a) Designed or helped to design a behaviour change intervention(s) that used specific behaviour change techniques (BCTs)?

☐ Extensively ☐ To Some Extent ☐ Not at all

(b) Used a taxonomy of BCTs (e.g. BCT Taxonomy v1) to code, design, or evaluate a behaviour change intervention?

☐ Extensively ☐ To Some Extent ☐ Not at all

(c) Designed or helped to design a behaviour change intervention(s) that was specifically grounded in a behaviour change theory/theories?

☐ Extensively ☐ To Some Extent ☐ Not at all

(d) Published papers/manuals/protocols of interventions that specify component BCTs?

☐ Extensively ☐ To Some Extent ☐ Not at all

(e) Published papers/manuals/protocols of interventions that specify behaviour change theory/theories?

☐ Extensively ☐ To Some Extent ☐ Not at all

(f) Undertaken a narrative or systematic review of behaviour change literature specifying interventions in terms of BCTs?

☐ Extensively ☐ To Some Extent ☐ Not at all

(g) Undertaken a narrative or systematic review of behaviour change literature specifying interventions in terms of behaviour change theory?

☐ Extensively ☐ To Some Extent ☐ Not at all

---

2. For approximately how many hours have you used BCT methodology? (e.g. 0-5, 10-20, 25-50, 50-100, 100+) ________

3. For how many studies have you used BCT methodology? ________

4. For how many studies have you explicitly used behaviour change theory? ________

5. How would you rate your knowledge/expertise in the following (where 0 = no knowledge/expertise and 7 = profound knowledge/expertise):

Behaviour change theories	0	1	2	3	4	5	6	7
Behaviour change techniques	0	1	2	3	4	5	6	7
Behaviour change interventions (evaluation or design)	0	1	2	3	4	5	6	7

6. Other relevant experience (please specify):

7. Please provide your contact details:

Name:

Email:
